# Immune activation and regulatory T cells in *Mycobacterium tuberculosis* infected lymph nodes

**DOI:** 10.1186/s12865-018-0266-8

**Published:** 2018-11-08

**Authors:** Karima Sahmoudi, Hassan Abbassi, Nada Bouklata, Mohamed Nouredine El Alami, Abderrahmane Sadak, Christopher Burant, W. Henry Boom, Rajae El Aouad, David H. Canaday, Fouad Seghrouchni

**Affiliations:** 1 Laboratory of Cellular Immunology, National Institute of Hygiene, 27, Avenue Ibn Batouta, PB 769, 11400 Rabat, Morocco; 2 Faculty of Sciences, University Mohammed V Agdal, Rabat, Morocco; 3 Department of ENT, Maxillo- facial, Reconstructive and Plastic Surgery, University Hospital Hassan II, Fes, Morocco; 4 National Reference Laboratory of Mycobacteriology, the National Institute of Hygiene, Rabat, Morocco; 5 Case Western Reserve University School of Nursing, Cleveland, USA; 6 TB Research Unit and Division of Infectious Diseases, Case Western Reserve University, University Hospitals of Cleveland and Cleveland VA, Cleveland, OH USA

**Keywords:** Tuberculosis, Lymphadenitis, Lymph node, Treg cells, conventional T cell, activation

## Abstract

**Background:**

Lymph node tuberculosis (LNTB) is the most frequent extrapulmonary form of tuberculosis (TB). Studies of human tuberculosis at sites of disease are limited. LNTB provides a unique opportunity to compare local *in situ* and peripheral blood immune response in active *Mycobacterium tuberculosis* (Mtb) disease. The present study analysed T regulatory cells (Treg) frequency and activation along with CD4+ T cell function in lymph nodes from LNTB patients.

**Results:**

Lymph node mononuclear cells (LNMC) were compared to autologous peripheral blood mononuclear cells (PBMC). LNMC were enriched for CD4+ T cells with a late differentiated effector memory phenotype. No differences were noted in the frequency and mutifunctional profile of memory CD4+ T cells specific for Mtb. The proportion of activated CD4+ and Tregs in LNMC was increased compared to PBMC. The correlation between Tregs and activated CD4+ T cells was stronger in LNMC than PBMC. Tregs in LNMC showed a strong positive correlation with Th1 cytokine production (IL2, IFNγ and TNFα) as well as MIP-1α after Mtb antigen stimulation. A subset of Tregs in LNMC co-expressed HLA-DR and CD38, markers of activation.

**Conclusion:**

Further research will determine the functional relationship between Treg and activated CD4+ T cells at lymph node sites of Mtb infection.

**Electronic supplementary material:**

The online version of this article (10.1186/s12865-018-0266-8) contains supplementary material, which is available to authorized users.

## Background

*Mycobacterium tuberculosis* (Mtb) infection is a major global health problem with approximately 10.4 million cases and 1.4 million deaths from tuberculosis (TB) in 2015 [1]. Furthermore, one-third of the world’s population is thought to be infected by Mtb. Extra pulmonary TB represents approximately 20% of clinical TB disease. Lymph node tuberculosis (LNTB) is the most frequent extrapulmonary form [1].

Cellular immune responses play a pivotal role in control of Mtb infection with CD4+ T cells having the central role. After infection CD4+ T cells undergo activation manifested by expression of surface molecules including HLA-DR and CD38 [2, 3]. Functionally, CD4+ T cells control infection by producing Th1 and Th17 cytokines [4]. Polyfunctional T cells, defined by their ability to co-express more than one cytokine, have been associated with protection against Mtb disease [4–6].

At sites of infection, immune responses are modulated by T regulatory cells (Tregs) [7, 8]. Tregs express CD3, CD4, high levels of CD25, low levels of the IL-7 receptor α-chain (CD127) and the intracellular marker forkhead box p3 (FoxP3) [9].

The relationship between Tregs and immune activation at sites of Mtb disease is not clear [10, 11]. The objective of the present study was to evaluate the interaction between Tregs and the function and activation of CD4+ T cells in lymph node vs. the peripheral blood compartments in persons with LNTB.

## Methods

### Subjects and preparation of immune cells

Eighteen patients (5 men, 13 women, age range 17-60 years) were recruited in the Hassan II University Hospital of Fes (Morocco) among patients with cervical lymphadenitis. Active LNTB was diagnosed by history, physical examination, and lab studies by experienced clinicians. The diagnosis of LNTB was based on a combination of clinical symptoms, pathology and response to TB drug therapy. Clinical symptoms associated with lymphadenitis included local lymphadenopathy, weight loss, fever, sweats, and anorexia. Histopathological evidence consisted of the presence of a granulomatous lesion with caseation in excisional biopsy specimens. Pulmonary radiography and HIV serology were performed to exclude pulmonary TB and HIV infection respectively. All LNTB cases were newly diagnosed and none had received anti-TB chemotherapy before sample collection. Tuberculin skin test results were positive (induration > 10 mm) for 15 out of 18 patients (83%). All patients were BCG vaccinated, and none reported contact with a case of pulmonary TB.

For all patients, the affected lymph node was in the neck and was surgically removed. In addition, 10 ml of peripheral blood was collected before starting anti-TB treatment. One portion of the lymph node was used for histological examination, and the other for isolation of lymph node mononuclear cells (LNMC) for immunologic studies.

Biopsy specimens were crushed gently in tissue culture medium. LNMC were spun and separated using Ficoll-Hypaque density centrifugation. Peripheral blood mononuclear cells (PBMC) were isolated from heparinized venous blood under endotoxin-free conditions by Ficoll-Hypaque (SIGMA) density centrifugation. Cells were cryopreserved and stored in liquid nitrogen until shipment by a cryoshipper to Case Western Reserve University for immunological studies.

### Phenotypic and functional study of T cells

PBMC and LNMC (10^6^/ tube) were stimulated with a pool of 34 overlapping peptides from Mtb-antigen ESAT6/CFP10 at 6.25 ug/ml per peptide (New England peptide, Gardner, MA), *M. tuberculosis* CDC1551 whole cells lysate (Mtb lysate) (BEI Resources) or staphylococcal enterotoxin B (SEB, 2 μg/ml, Sigma) overnight at 37 °C in 5% CO_2_. Unstimulated PBMC and LNMC served as negative controls. Anti-CD28/CD49d (1 μg/ml each, eBioscience and Biolegend) was added to each tube during stimulation and brefeldin A (5 μg/ml, Sigma) was added 2 hr later. After stimulation, cells were washed with PBS and surface stained with anti-CCR7-PE-Cy7 (BD Bioscience) for 15 min at 37 °C, then live dead yellow (Invitrogen), anti-CD14-BV570, anti-CD4-APC/Cy7, anti-CD8-BV510 (all Biolegend) and anti-CD45RA-PE/TR (Invitrogen) were added and incubated at RT for 25 min. Afterward cells were washed, permeabilized (Cytofix/Cytoperm Kit, BD Pharmingen) according to the manufacturer’s instructions and stained for intracellular expression with anti-CD3-PerCP, anti-IFNγ-Alexa700, anti-IL2-APC, anti-TNFα- Pacific Blue, anti-IL17-BV711 (all Biolegend) and anti-MIP-1α-FITC (R&D). Cells were then washed, fixed in 1% paraformaldehyde and 1x10^6^ total events collected from each sample on an LSR-II flow cytometer (BD). Net responding cells for each cytokine were calculated by subtracting the no antigen condition (medium only) from the antigen simulated result. The analysis of all functional markers expressed after stimulation were done on viable total memory CD4+ and CD8+ T cells. Memory phenotype was determined by CCR7 and CD45RA expression. Naïve T cells were CD45RA+/CCR7+, central memory (CM) cells were CD45RA-/CCR7+, effector memory (EM) cells were CD45RA-/CCR7-and effector cells (E) were CD45RA+/CCR7-.

To identify Tregs and activated T cells, PBMC and LNMC were surface stained with live dead yellow (Invitrogen), anti-CD3-APC/Cy7, anti-CD4-PerCP, anti-CD8-Alexa700, anti-CD25-APC, anti-HLA-DR-FITC, anti-CD38-PE/Cy7(all Biolegend), and anti-CD127-BV650(BD Bioscience), and incubated at RT for 25 min. After, cells were washed, permeabilized and intracellular stained with, anti-FoxP3-PE (eBioscience) or isotype-matched negative control for gating purposes.

Plots were analyzed using FlowJo software (version 6.1.1; Tree Star, Ashland, OR, USA). Boolean analysis on Flow Jo and SPICE (NIAID, NIH, USA) was used to assess cytokine polyfunctionality.

### Statistical analysis

Statistical analysis was performed using the paired Student’s t test for comparisons of PBMC and LNMC. P value for comparison of polyfunctional profile was done using SPICE software. Significance of correlations was analyzed by the nonparametric Spearman test. A *p* value of < 0.05 was considered significant. Data were analyzed by Statistical Package for the Social Sciences (SPSS) software (IBM) and the GraphPad Prism software, version 5.00 for Windows (GraphPad Software, San Diego).

## Results

### Phenotypic and cytokine profile of CD4+ T cells in LNMC and PBMC

**Fig. 1:**
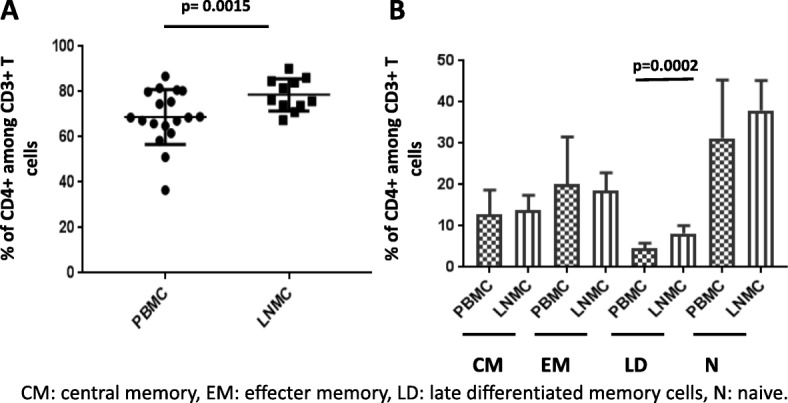
Phenotypic and functional T-cell subset distribution in PBMC and LNMC. Relative frequencies of CD4+T cells (**a**) and memory subsets of CD4+ T cells (**b**) among all CD3+ T cells. Means from 18 subjects are shown and error bars represent standard deviations

We first compared memory phenotypes of T lymphocyte subsets in LNMC and PBMC. LNMC were significantly enriched for CD4+ T cells [80.7±5.6 % vs. 68.0±6.6 % (*p* = 0.0015)] (Fig. 1a). CD8+ T cells were concomitantly lower in LNMC compared to PBMC [17.6±5.8 % vs. 27.4±11.7 % (*p* = 0.0055)] (Additional file 1). Memory subsets of CD4+ T cells were defined according to expression of CD45RA and CCR7 and only the effector subset was modestly increased in LNMC compared to PBMC (*p* = 0.0002) (Fig. 1b).

**Fig. 2:**
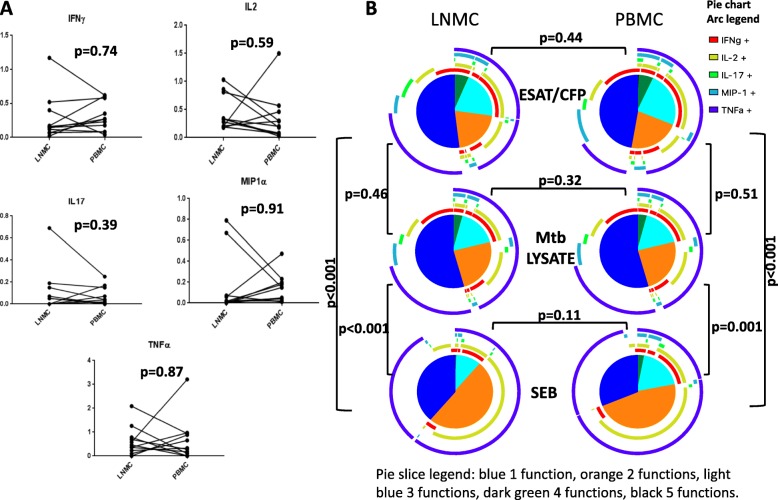
Cytokine expression of memory CD4+T cells after Mtb antigen RVL stimulation (**a**) and polyfunctional composition of total memory CD4+T cells after stimulation with Mtb lysate, ESAT6/CFP10, and SEB (**b**). Results from 11 subjects are shown. Plots are gated on viable memory CD4+T cells. The pie charts depict the average polyfunctional profile of only the responding cells for each specific stimuli from all subjects. All possible combinations of responses are represented by the arcs with pie arc color legend on the figure

T cell functional profiles in response to Mtb antigens were compared between LNMC and PBMC. IFNγ, IL2, TNFα, IL17, and MIP-1α production by CD4+ T cells in PBMC and LNMC in response to Mtb RVL were not different (Fig. 2a). Similarly CD4+ T cell responses in response to ESAT6/CFP10 peptides were not different between LNMC and PBMC (Additional file 2).

No significant differences were observed in the polyfunctionality of total memory CD4+ T cells in LNMC when compared to PBMC for the same stimuli (Fig. 2b all three rows with side to side comparison). Polyfunctionality of total memory CD4+ T cells in response to ESAT6/CFP10 peptides and Mtb lysate were significantly different from that in response to SEB in both PBMC and LNMC indicating a difference in Mtb-specific cells as a group (Fig. 2b, top 4 pies vs. bottom 2 comparison), but no differences were found in the polyfunctionality in response to Mtb-specific antigens, ESAT6/CFP10 peptides and Mtb lysate (Fig. 2b top two and middle two comparison).

### Distribution of activated T cells and Tregs in LNMC and PBMC

**Fig. 3:**
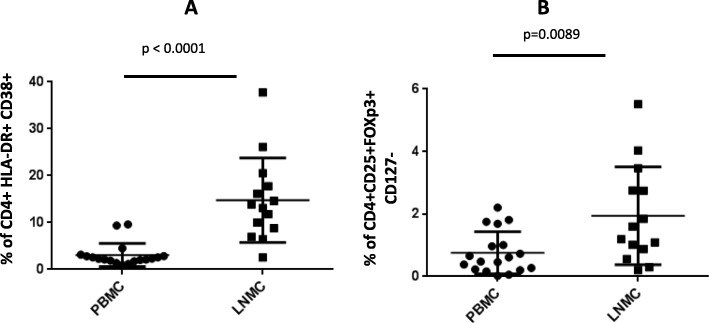
Frequency distribution of HLA-DR+ CD38+ activated (**a**) and FoxP3+CD25+CD127- (**b**) regulatory CD4+T cells in LNMC and PBMC

**Fig. 4:**
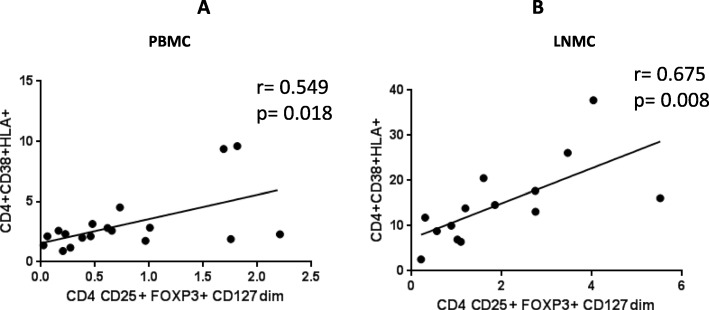
Ex vivo frequencies of Tregs correlated with the ex-vivo frequencies of CD4+ activated cells in PBMC (**a**) and LNMC (**b**)

We measured the frequency of activated conventional CD4+ T cells and Tregs in LNMC and PBMC. Activation was based on the expression of HLA-DR and CD38. Tregs were identified as CD4+, CD25+, FoxP3+ and CD127dim. An increased proportion of activated CD4+ HLA-DR+CD38+ T cells (*p* < 0.0001) and Treg CD4+ CD25+FoxP3+CD127dim (*p* = 0.0089) in LNMC compared to PBMC was observed (Fig. 3). A positive correlation between the frequency of total activated CD4+ T cells and Tregs was observed within LNMC (*r* = 0.676, *p* = 0.008) and PBMC (*r* = 0.549, *p* = 0.018) (Fig. 4).

**Fig. 5:**
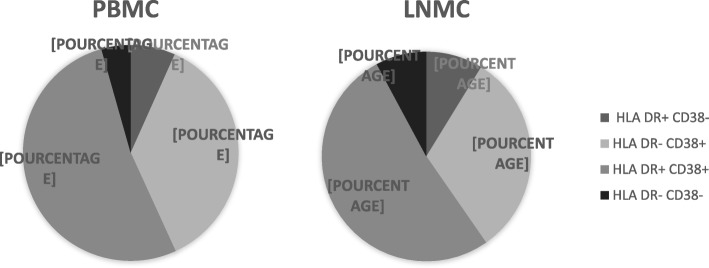
Distribution of activation markers within CD4+CD25+FoxP3+CD127dim Tregs in PBMC and LNMC

We compared, among Tregs in LNMC, the proportion of cells expressing activation markers. More than 90% of CD25+ FoxP3+CD127dim cells expressed HLA-DR and/or CD38. Furthermore, the predominant Treg subpopulation in both LNMC and PBMC was the one expressing HLA-DR and CD38 simultaneously (Fig. 5).

### Treg correlation with Mtb-antigen induced cytokine production

We determined the correlation between the proportion of Tregs and cytokine producing CD4+ T cells after overnight Mtb-antigen activation in LNMC and PBMC. There was a positive correlation between the proportion of Tregs and Th1 cytokine production (IL2 and IFNγ) as well as MIP-1α following stimulation by Mtb lysate antigen within LNMC but not among PBMC (Table 1). A similar correlation was found after stimulation by Mtb ESAT6/CFP10 peptides while no correlation was found after SEB mitogen stimulation (data not shown).

**Table 1:** Correlation of the ex vivo frequencies of Tregs with the frequencies of CD4+T cells expressing IFNγ, IL2, TNFα, IL17 and MIP1α following Mtb lysate antigen stimulation in PBMC and LNMC

	T reg			
	PBMC		LNMC	
	*r*	*p*	*r*	*p*
IFN-γ	-0.19	0.60	0.66	0.04
IL2	-0.04	0.90	0.71	0.02
TNF-α	-0.02	0.96	0.48	0.16
IL17	-0.23	0.52	0.45	0.12
MIP-1α	-0.04	0.91	0.95	<0.001

## Discussion

The outcome of the immune response to Mtb results from the simultaneous involvement of activating and regulatory mechanisms [8, 11]. The nature of the relationship between conventional T cells and Tregs during active TB is still not clear although this interaction has been studied in different forms of TB [10, 11]. Increased expression of activation markers on T cells [12, 13] and higher levels of Tregs [7, 8] have been described in peripheral blood of patients with TB. Nevertheless, local immune responses may differ from those in peripheral blood and exploring this interaction in the site of active infection will give important clues about their involvement in protection or pathogenesis [14, 15]. The present study sought to evaluate the relationships between Tregs and conventional CD4+ T cells in lymph nodes and peripheral blood during TB lymphadenitis.

We found a higher proportion of CD4+ T cells in LNMC compared to PBMC. This is in agreement with previous studies reporting an increase in the proportion of CD4+ T cells in the blood of TB patients compared to uninfected controls and a much higher number of CD4+ T cells at the site of infection [16, 17]. Except for modest elevation in effector cells in LNMC, no difference was found in the relative frequency of memory CD4+ T cell subsets between PBMC and LNMC. Others have shown that Mtb-specific CD4+ T cells in bronchoalveolar and pleural fluids are mainly of the memory phenotype [18, 19]. In LNMC we find that up to 40% of CD4+ T cells were naïve.

Th1 and Th17 Mtb–specific T cells contribute to the defense against progressive Mtb infection. Particularly, TB lymphadenitis was characterized by elevated frequencies of Th1 and Th17 cells in peripheral blood [20]. When we measured intracellular production of Th1 cytokines (IFNγ, IL2 and TNFα), IL17 and MIP-1α, we were surprised to not find an increase in the frequencies of Mtb-specific cytokine producing cells in LNMC vs. PBMC. LNMC were generated from a large block of excised lymph node tissue that included granulomatous and non-granulomatous areas which may have affected our ability to detect higher frequencies of Mtb-specific cells in LNMC than PBMC.

Polyfunctional T cells are correlated with protection in some studies [5, 6, 21] and with disease activity in others [22, 23]. In our case, no differences were observed in the proportion and polyfunctional qualities of the Mtb-responsive CD4+ T cells between LNMC and PBMC. Comparing polyfunctionality in CD4+ T cells responding to Mtb-antigen vs. SEB showed a significant difference with SEB eliciting a more TNFα dominated response. This supports that the Mtb-specific responses were different from those of the whole memory CD4+ T cell pool.

To analyze the interaction between conventional activated CD4+T cells and Tregs in the lymph node during active TB, we measured the frequency of CD4+T cells expressing CD38 and HLA-DR. These immune activation markers were described as substantially elevated in subjects with active TB [24]. CD4+ T cells were more activated in lymph node compared to blood. The increased proportion of activated T cells in LNMC likely reflects more exposure to Mtb antigens. The selective accumulation is likely the result of both active recruitment and local expansion of T cells at this site of Mtb replication [25, 26].

Tregs were identified by selecting CD4+ cells with high-level expression of CD25, low-level expression of CD127, and expression of FoxP3. Tregs were also increased in LNMC in response to local immune activation possibly to control immune induced damage [3, 26, 27]. In accordance with the previous study [10], our data reveal a positive correlation between Treg and CD4+ activated cells within PBMC but this correlation was stronger in LNMC. Tregs co-expressing HLA-DR and CD38 markers were frequent in the LNMC and PBMC. The ex-vivo MHC II expressing Tregs are believed to be a functionally mature distinct Treg subset implicated in contact-dependent *in vitro* suppression [28]. A positive correlation between Treg proportion and frequency of memory Mtb-specific CD4+ T cells expressing each individual measured Th1 function was observed in LNMC but not in PBMC. The predominance of activated Tregs in LNMC and the correlation of Tregs with activated CD4+ T cells and CD4+ T cells expressing Th1 cytokines suggests a regulatory activity specific and enhanced in the lymph node. An alternate hypothesis could be that the Treg are just expanding at tissue sites but not necessarily for regulatory purposes. The absence of an expected higher proportion of Th1 producing CD4+T cells in LNMC compared to PBMC supports the hypothesis that the Treg/CD4 correlation reflected a negative feedback for excess Th1 cytokine production by increasing suppressive Tregs. Recent data suggest that IFNγ increases Treg suppressive function for control of Th1 responses [29, 30]. A limitation of the study is that we did not have any blood or lymph node material in healthy BCG-vaccinated or latently infected individuals available to contrast to the TB lymphadenitis subjects. Contrasting these cohorts in future studies as well as further research on the function of Tregs and their modulation of immune responses in TB lymphadenitis are needed. The current study will help develop an optimal approach to such supplementary exploration.

## Conclusion

This study has important original findings regarding local immune responses in active LNTB. We found no Th1, Th17 or MIP-1α production differences by Mtb-specific CD4+ T cells in total LNMC vs. PBMC. Tregs were positively correlated with Th1 expressing Mtb-specific CD4+ T cells in LNMC but not in PBMC. Activated HLA-DR+CD38+Tregs were more abundant, suggesting modulation by Tregs of immune responses in LNMC. We suggest that increased Tregs at the lymph node site of active Mtb infection regulates the immune response. Whether this is advantageous to the host or not remains to be determined.

## Additional files

Additional file 1: CD8+ T-cell subset distribution in PBMC and LNMC. Relatives frequencies of CD8+ T cells among all CD3+ T cells. Means from 18 subjects are shown and error bars representing standard deviations. (PPTX 51 kb)

Additional file 2: Cytokine expression of memory CD4+T cells after ESAT6/CFP10 stimulation. Results from 11 subjects are shown. Plots are gated on viable memory CD4+T cells. (PPTX 91 kb)

## Abbreviations

LNTB: Lymph node TB
TB: Tuberculosis
Mtb: Mycobacterium tuberculosis
Treg: T regulatory cells
LNMC: Lymph node mononuclear cells
PBMC: Peripheral blood mononuclear cells
CD127: IL-7 receptor α-chain
FoxP3: The intracellular marker forkhead box p3
Mtb lysate: M. tuberculosis CDC1551 whole cells lysate
SEB: Staphylococcal enterotoxin B
N: Naïve T cells
CM: Central memory cells
EM: Effector memory cells
E: Effector cells
