# Supplementary figures and images for: Immune activation and regulatory T cells in Mycobacterium tuberculosis infected lymph nodes
Source: BMC Immunol. 2018 Nov 8;19:33. doi: 10.1186/s12865-018-0266-8 (PMC6225640; doi:10.1186/s12865-018-0266-8)

## Slide 1
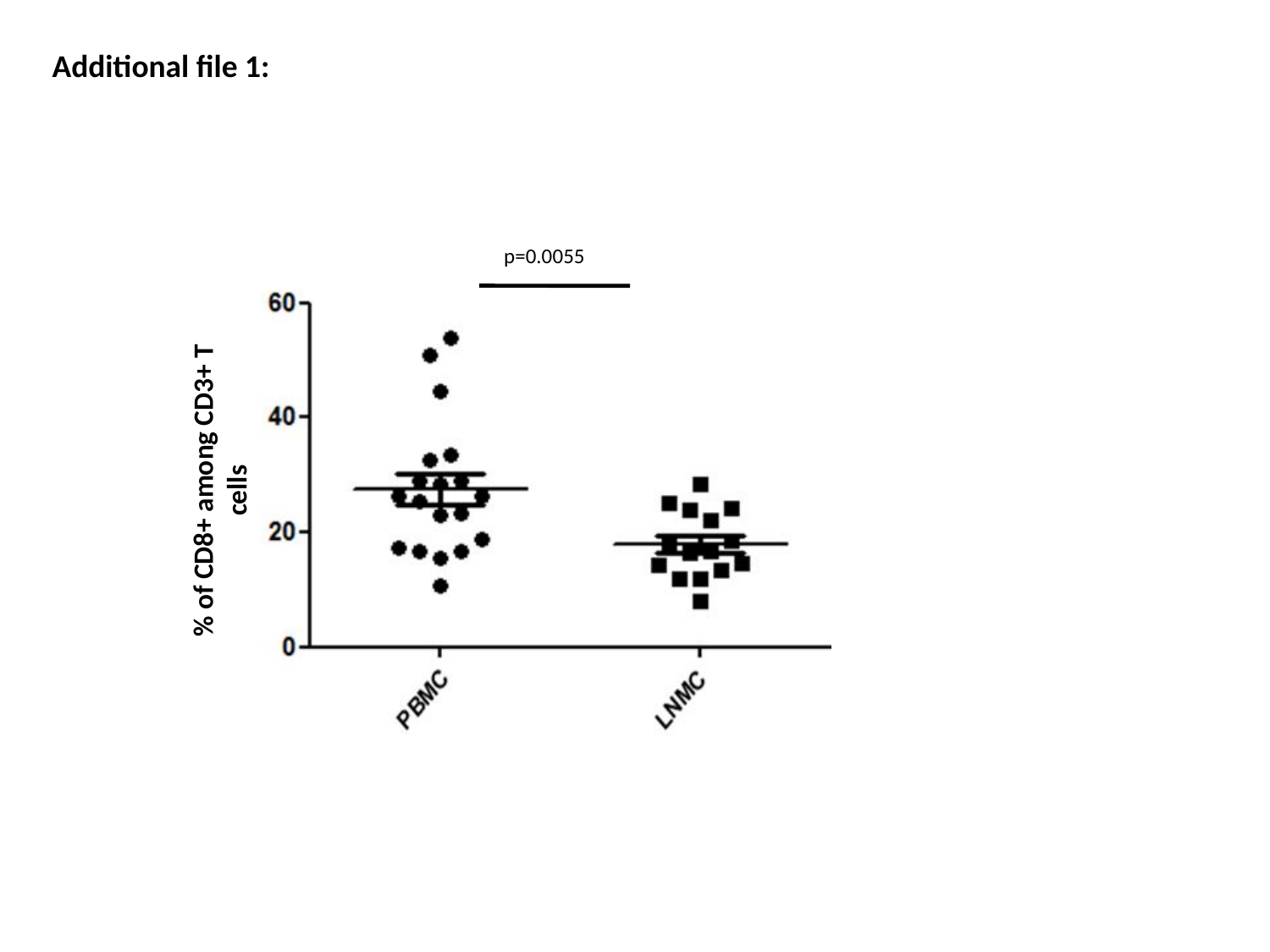

Additional file 1:
p=0.0055
% of CD8+ among CD3+ T cells

Supplement: Supplementary file 1 — CD8+ T-cell subset distribution in PBMC and LNMC. Relatives frequencies of CD8+ T cells among all CD3+ T cells. Means from 18 subjects are shown and error bars representing standard deviations. (PPTX 51 kb) [file 12865_2018_266_MOESM1_ESM.pptx]

## Slide 1
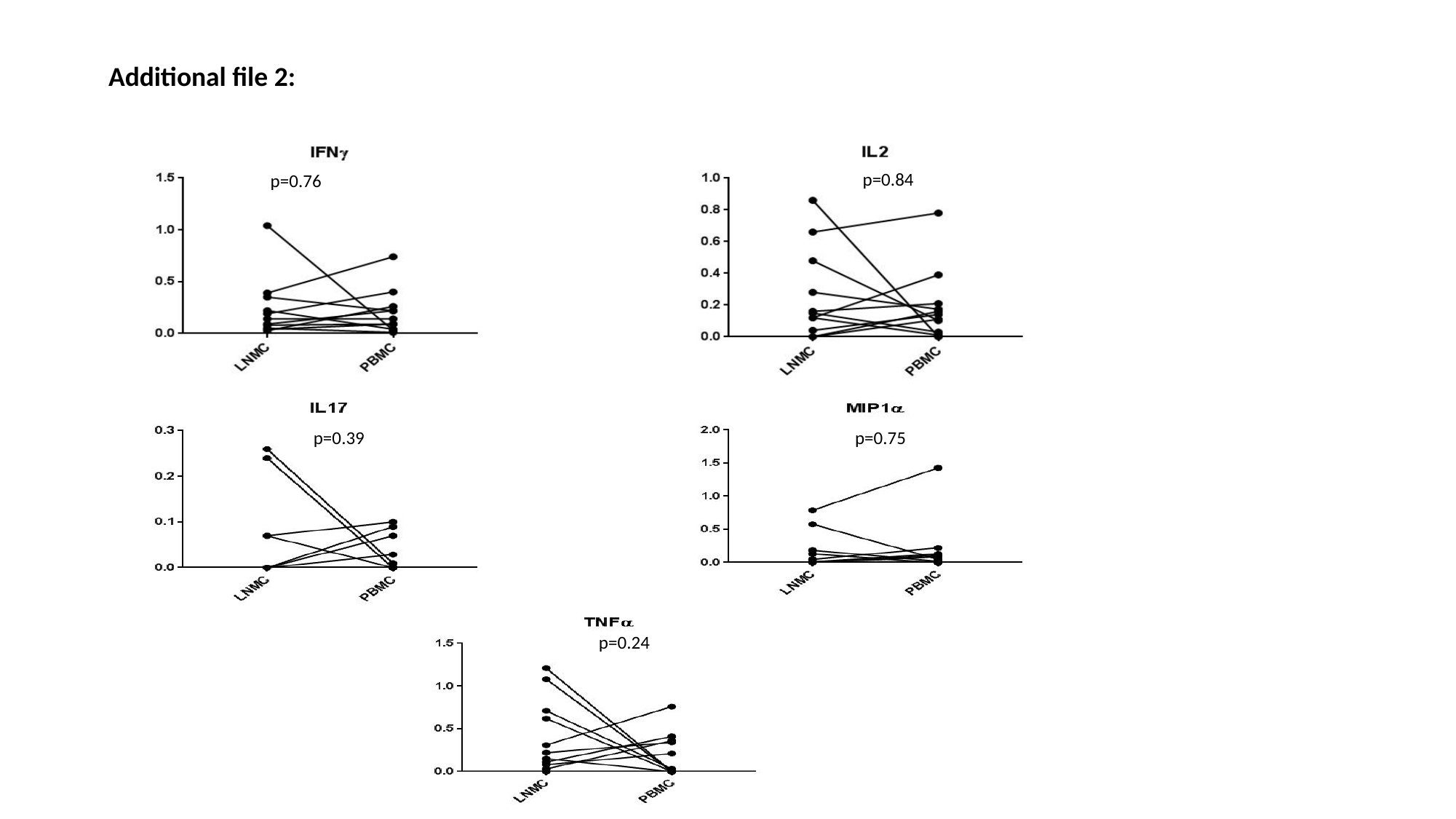

Additional file 2:
p=0.84
p=0.76
p=0.39
p=0.75
p=0.24

Supplement: Supplementary file 2 — Cytokine expression of memory CD4+T cells after ESAT6/CFP10 stimulation. Results from 11 subjects are shown. Plots are gated on viable memory CD4+T cells. (PPTX 91 kb) [file 12865_2018_266_MOESM2_ESM.pptx]
